# The Red Light Treatment of Small-Pox

**Published:** 1903-07-25

**Authors:** 


### THE RED LIGHT TREATMENT OF SMALL-POX.

As cases of small-pox are fairly common just now in some parts of England it is worth while to consider the claims of the light treatment of this disease. Finsen [1] considers that from various trials which he has made and which he says were duly controlled by simultaneous experiments he is justified in concluding that sunlight, and especially its chemical rays, have a most injurious effect on the course of small-pox; and he goes so far as to ascribe the suppuration of the vesicles entirely to the effect of light. In consequence of this he claims that where it is possible to protect the patient during his illness from the action of ordinary light, it is possible also to protect him from suppuration and the consequences thereof. He does not claim that light has any effect on the small-pox infection itself, but states very firmly that it does most distinctly influence the vesicular pustulation.

It must be admitted that even with this limitation the advantages of the treatment would be very great, for the suppuration stage is certainly one of the most dangerous of the several stages through which small-pox passes it may probably be said with truth that the greater number of small-pox deaths are due to complications and sequelæ brought about by the suppuration. Moreover, absence of pustulation means, of course, to a great extent absence of pitting and consequent disfigurement, and this, again, is no small consideration.

Professor Finsen is so strong in his opinion that in a recent number of the *British Medical Journal* he has expressed the view that it is "absolutely unwarrantable on the part of the public health authorities to treat serious cases of small-pox in which suppuration may be expected in hospitals where the patients are exposed to light," and that as to the private physician "it must be deemed a gross shortcoming on his part if, as soon as he diagnoses small-pox, he does not make preparations to prevent the patient from being exposed to daylight." A physician, he says, "who allows a patient with small-pox to lie in daylight, shows the same stupendous incompetence as would a surgeon who neglected to take ordinary aseptic measures in performing a capital operation."

These are strong words and if justified it is strange that in the ten years that have elapsed since first Professor Finsen advocated the "dark" treatment of small-pox, the matter has not been more thoroughly threshed out. To do so would be comparatively simple, for it is to be remembered that the light to which Professor Finsen refers and against which he inveighs is merely that of the blue, violet, and ultra-violet end of the spectrum. All that is necessary therefore to meet his requirements is either to keep the patient in entire darkness, inspecting him by the occasional light of a candle, or to cover with red curtains windows and all other places through which ordinary light may enter; in short to turn the sick chamber into a large "photographer's room."

In support of Finsen's contention it is to be remembered that he is not alone in his belief that the chemical rays of sunlight are productive of cutaneous changes of an inflammatory nature. Unna, Maklakoff,[2] Charcot,[3] and others have all produced evidence upon this point, and Finsen's pretension is that while all skins are more or less affected by sunlight, that of a small-pox patient is as sensitive as a photographic plate. Long before Finsen began his advocacy of red light, others had suggested allied treatment even in quite early times. Not to go too far back Black in 1867 and Waters in 1871 may be mentioned. Since Finsen took the matter up he has won many disciples in Norway, while in Germany and France the method has been tried and reported on very favourably by several observers. It is a matter, however, on which self-deception is very easy, for it is impossible to predicate in any given case whether suppuration will occur or whether it will not. The most threatening cases sometimes dry up unexpectedly without special measures, and hence it is not easy to put the question to very conclusive proof. Oetlinger,[4] who made an exceedingly careful study of the treatment, pointed this out in 1894, but nevertheless is upon the whole in favour of its general adoption. Although three out of nine severe cases which he specially selected for trial died, the results in the remaining six were decidedly good. The most recent writer on the subject is Schamberg, who tried it in two cases, with results which in his opinion were entirely unfavourable to it. He was unable to see that the course of the disease was modified in any way whatever, though he admits that two cases only are scarcely sufficient from which to draw conclusions. He considers that Finsen and others have been misled by the type of the disease with which they have had to deal. He also points out that Denmark and Sweden and Norway are among the best vaccinated countries in Europe, and that the vast majority of persons who have once been vaccinated recover without scarring under any or no treatment, when they recover at all. There is some truth in this, but nevertheless the treatment can be so readily undertaken, that it may be worth while to give it an extended trial. The views of a man like Finsen cannot be lightly put aside, and the epidemic during which Œtlinger made his experiments was certainly of a severe type, while it is obvious that he examined the claims of the treatment without any pre-existing bias in its favour. Both he and Finsen, it is to be noted, make a strong point of the treatment being early adopted if success is to be ensured.

[1] Brit. Med. Jour., June 6, 1902. [2] Compter Rendu de la Soc. de Biol., 1889. [3] Archiv. d'Ophthalmologie, 1889, p. 397. [4] Semaine Medicale, May 1894.

